# Decreased Efficiency of Very-Low-Density Lipoprotein Lipolysis Is Linked to Both Hypertriglyceridemia and Hypercholesterolemia, but It Can Be Counteracted by High-Density Lipoprotein

**DOI:** 10.3390/nu13041224

**Published:** 2021-04-08

**Authors:** Ewa Wieczorek, Agnieszka Ćwiklińska, Agnieszka Kuchta, Barbara Kortas-Stempak, Anna Gliwińska, Maciej Jankowski

**Affiliations:** Department of Clinical Chemistry, Faculty of Pharmacy, Medical University of Gdańsk, 80-210 Gdańsk, Poland; agnieszka.cwiklinska@gumed.edu.pl (A.Ć.); agnieszka.kuchta@gumed.edu.pl (A.K.); barbara.kortas-stempak@gumed.edu.pl (B.K.-S.); anna.gliwinska@gumed.edu.pl (A.G.); maciej.jankowski@gumed.edu.pl (M.J.)

**Keywords:** high-density lipoprotein, hypercholesterolemia, hypertriglyceridemia, lipolysis, lipoprotein lipase, triglycerides, very-low-density lipoprotein

## Abstract

Impaired triglyceride-rich lipoprotein plasma catabolism is considered the most important factor for hypertriglyceridemia development. The aim of this study was to evaluate the impact of hypercholesterolemia and hypertriglyceridemia on the efficiency of lipoprotein lipase (LPL)-mediated very-low-density lipoprotein (VLDL)-triglyceride lipolysis and the role of high-density lipoprotein (HDL) in this process. Subjects with no history of cardiovascular disease (CVD) and untreated with lipid-lowering agents were recruited into the study and divided into normolipidemic, hypercholesterolemic, and hyperlipidemic groups. VLDL was isolated from serum and incubated with LPL in the absence or presence of HDL. For the hypercholesterolemic and hyperlipidemic groups, a significantly lower percentage of hydrolyzed VLDL-triglyceride was achieved compared to the normolipidemic group (*p* < 0.01). HDL enhanced the lipolysis efficiency in the hypercholesterolemic and hyperlipidemic groups on average by ~7% (*p* < 0.001). The lowest electrophoretic mobility of the VLDL remnants indicating the most effective lipolysis was obtained in the normolipidemic group (*p* < 0.05). HDL presence significantly reduced the electrophoretic mobility of the VLDL remnants for the hypercholesterolemic and hyperlipidemic groups (*p* < 0.05). The results of our study indicate that VLDL obtained from hypercholesterolemic and hyperlipidemic subjects are more resistant to lipolysis and are additional evidence of the need for early implementation of hypocholesterolemic treatment, already in asymptomatic CVD subjects.

## 1. Introduction

Cardiovascular disease (CVD) is the leading cause of death worldwide [1]. The major lipid risk factor for CVD development is hypercholesterolemia [2], but accumulating evidence from recent studies shows that an increased triglyceride (TG) level is also strongly associated with cardiovascular morbidity and mortality risk [3].

Hypertriglyceridemia (HTG) occurs in approximately 27% of the population [4] and is closely linked to obesity, considered to be a pandemic of the 21st century [5]. The development of HTG can be related to genetic predisposition [6], excessive synthesis, and secretion of TG-rich lipoproteins (TRL), including chylomicron (CM) and very-low-density lipoprotein (VLDL) [7], and/or to impaired TRL clearance from plasma [8,9].

Impaired TRL clearance is the most important factor that contributes to the development of HTG, and the reduced efficiency of intravascular TG hydrolysis mediated by lipoprotein lipase (LPL) is crucial to this process [9]. The impaired clearance leads to a prolonged presence of TRL remnants in plasma that infiltrate into the subendothelial space, causing inflammation and endothelial dysfunction [10,11]. The increased number of TRL remnants has also been related to the induction of coagulation activity, the generation of highly atherogenic small dense low-density lipoprotein (sdLDL), and an impairment of high-density lipoprotein (HDL) composition and function [6].

HDL plays an important anti-atherogenic role [12], and recent studies show that the beneficial effect of HDL is linked to the functional traits of the lipoprotein rather than its quantity measured with cholesterol content [13]. Plasma TRL and HDL metabolism is closely related [12]: it has been shown that HDL protects the endothelium from the inhibitory effect of VLDL on endothelium-dependent relaxation [14]. The other important mechanism by which HDL may affect TRL metabolism and prevent HTG development and atherosclerosis as a consequence is the interaction between HDL and TRL particles. It has been previously shown that HDL acts as an acceptor of the surface material released from VLDL during LPL-mediated lipolysis, enhancing the efficiency of TG lipolysis. HDL also promotes the conversion of VLDL into IDL/LDL, and the changes in composition and properties of HDL related to metabolic disorders, such as chronic kidney disease, can impair this activity [15].

Since the metabolism of TG and cholesterol are linked due to the shared physicochemical properties of the molecules and their presence in the particles of the same lipoprotein classes, there may be a relationship between hypercholesterolemia and HTG development. For instance, it was demonstrated that subjects with familial hypercholesterolemia (FH) with normal fasting TG levels had impaired postprandial TG response to fatty meals compared to control, despite similar basal TG levels in FH and control groups [16,17]. Whether the oversecretion and/or impaired TRL catabolism and clearance can be the cause of TRL disturbances and HTG development in hypercholesterolemic subjects is not fully explained and needs to be elucidated [18].

The aim of this study was to evaluate the impact of hypercholesterolemia and hypertriglyceridemia on the efficiency of LPL-mediated VLDL lipolysis in subjects with no history of CVD to assess TG-rich lipoprotein metabolism disturbances. The magnitude of the effect of HDL on VLDL lipolysis efficiency was also assessed. We found that similarly to hypertriglyceridemic subjects, the VLDL obtained from hypercholesterolemic subjects with no increased TG serum level had reduced susceptibility to lipolysis compared to controls with normal lipid levels. HDL was able to increase lipolysis efficiency independently on lipid disturbances.

## 2. Materials and Methods

### 2.1. Study Groups

Forty adults aged 21–59 under the care of the General Practitioner (Non-public Health-Care Center, Pomerania region, Poland) were recruited into the study. Participants were asymptomatic for CVD at the time of recruitment, and a history of a previous cardiovascular event was excluded. The other detailed study exclusion criteria were: diabetes, liver disease, kidney disease, cancer, asthma, acute disease within 3 months before the study, a treatment course containing lipid-lowering drugs (statins, fibrates, ezetimibe, and PCSK9 inhibitors), hormone replacement therapy (HRT) and other drugs affecting lipid profile (immunosuppressive agents, diuretics, steroids), or heparin. All participants gave written informed consent for participation in the study. The study was approved by the Independent Bioethics Commission for Research of the Medical University of Gdansk, Poland (No. NKBBN/612/2017–2018).

Study participants were divided into three groups according to low-density lipoprotein cholesterol (LDL-C) and TG levels. Namely, LDL-C < 115 mg/dL and TG < 150 mg/dL, the normolipidemic (NL) group; LDL-C ≥ 115 mg/dL and TG < 150 mg/dL, the hypercholesterolemic (HC) group; and LDL C ≥ 115 mg/dL and TG ≥ 150 mg/dL, the hyperlipidemic (HL) group. The characteristics of the study groups are presented in Table 1. There were 12 subjects (30%) that were normolipidemic and 28 that had elevated serum lipid levels. There were 15 patients (38%) who had increased LDL-C levels (HC group) and 13 subjects (32%) who had both increased LDL-C and TG levels (HL group). There were no significant differences in BMI, gender distribution, hypertension, and smoking frequency among the groups.

### 2.2. VLDL and HDL Isolation

Vein blood was collected from participants after overnight fasting into commercially available test tubes (BD Vacutainer, Franklin Lakes, NJ, USA) to obtain the serum. The VLDL was isolated by ultracentrifugation as described previously [19]. Briefly, 1.8 mL of serum were transferred into a 3.2 mL ultracentrifuge tube (Polyallomer Bell-top, Beckman Coulter, Brea, CA, USA) and gently overlaid with NaCl (d = 1.006 g/mL). The ultracentrifugation was performed in a Beckman Optima TM TLX Ultracentrifuge using a Beckman fixed-angle rotor (TLA 100.3) for 60 min, at 541,000× *g*, 4 °C, acceleration 0, deceleration 9. After ultracentrifugation, the VLDL was collected by tube slicing (Beckman Tube Slicer). The HDL was isolated from infranatant after VLDL isolation by a precipitation and ultracentrifugation method described by McPherson [20] with modification. Briefly, the HDL in the infranatant was separated from apo B containing lipoproteins by precipitation with heparin (5000 U/mL) (Polfa Warszawa, Warsaw, Poland) and 1 M MnCl_2_. Next, 2.5 mL HDL was adjusted to density 1.21 g/mL by adding solid KBr. Density adjusted HDL was transferred into a 3.2 mL ultracentrifuge tube (Polyallomer Bell top, Beckman Coulter, Brea, CA, USA), and the respective volume of an aqueous solution of KBr (with a density of 1.21 g/mL) was added. Ultracentrifugation was performed in a Beckman Optima TM TLX Ultracentrifuge using a Beckman fixed-angle rotor (TLA 100.3) for 150 min, at 541,000× *g*, 4 °C. After ultracentrifugation, 1.9 mL of supernatant containing the HDL fraction was collected by tube slicing.

The VLDL and HDL were dialyzed against 0.01 M Tris-HCl buffer, pH 7.4, containing 0.195 M NaCl and 0.01% NaN_3_ (*w*/*v*) as a preservative for 18 h at 4 °C [15] and they were used in the lipolysis study.

### 2.3. VLDL Lipolysis Study

The VLDL lipolysis was performed according to the procedure described earlier [15]. Briefly, VLDL was incubated with LPL isolated from bovine milk (Sigma Aldrich, St. Louis, MO, USA) for 1 h at 37 °C at a constant VLDL-TG:LPL 90:0.48 mg/dL ratio, in the absence or presence of HDL. The VLDL-cholesterol:HDL-cholesterol weight ratio was 1:1. Albumin (Sigma Aldrich, St. Louis, MO, USA) was added to the reaction mixtures as a free fatty acid acceptor (final concentration in the reaction mixture: 2% *w*/*w*). An appropriate volume of 0.01 M Tris‑HCl buffer, Ph 7.4, containing 0.195 M NaCl and 0.01% NaN_3_ corresponding to the volume of HDL and/or LPL was added to adjust the differences between reaction mixtures volumes. The control mixture (called “VLDL control”) was incubated with VLDL and albumin, without LPL and HDL, and with an appropriate volume of 0.01 M Tris-HCl buffer, Ph 7.4, containing 0.195 M NaCl and 0.01% NaN_3_. After incubation, the mixtures were cooled on ice for 10 min. The VLDL remnants were separated from other reaction products by immunoprecipitation with anti-apo B polyclonal antibodies (Dako, Glostrup, Denmark) (reaction mixture:antibodies 2:1 (*v*/*v*) ratio) [21], and the concentration of hydrolyzed TG was measured.

### 2.4. Biochemical Analysis and Electrophoresis

The lipid concentration of TG, total cholesterol (TC), phospholipids (PL) in serum, VLDL, and HDL was measured using commercially available enzymatic kits obtained from Pointe Scientific, Poland (TG, TC) and Wako Diagnostics, USA (PL). The LDL-C concentration was calculated using the Friedewald formula. The apolipoprotein (apo) AI, B, CII, CIII, E concentration was measured by immunonephelometry using kits obtained from Siemens Healthcare, Germany (apo AI, apo B, apo E) and Randox, Poland (apo CII, apo CIII). Lipoprotein agarose electrophoresis was carried out using a commercially available Hydragel 7 LIPO + Lp(a) kit obtained from Sebia (Lisses, France). Densitometric analysis of electropherograms was performed with Launch VisionWorksLS (Thermo Fisher Scientific, Waltham, MA, USA). The relative electrophoretic mobility of the lipoproteins was calculated by dividing the distance of the lipoprotein band measured from the sample application site by the distance of the dye front measured from the sample application site after 1.5 h of separation.

### 2.5. Statistical Analysis

Statistical analysis was performed using STATISTICA 13 software (StatSoft Polska Sp. z. o.o., Cracow, Poland). Normality was assessed with the Kolmogorov–Smirnov test. The categorical data are presented as numbers and percentages, and a chi-squared test was used to compare the groups. The continuous data are presented as mean ± standard deviation (SD) or median (twenty-fifth–seventy-fifth percentiles). The difference between the study groups was assessed using the analysis of variance (ANOVA) with Tukey post-hoc test or Kruskal–Wallis with Dunn’s post-hoc test. In lipolysis efficiency studies, the differences between the groups were assessed using analysis of covariance (ANCOVA) with a post-hoc Bonferroni test considering VLDL-TG concentration in reaction mixtures as a covariate since TG levels are a significant variable affecting lipolysis efficiency [15]. Age was used as an additional covariate because there was a relationship between lipolysis level and age. Lipid and apolipoprotein VLDL components were not included in the analysis since they were closely correlated with VLDL-TG level. The correlation between the variables was analyzed by Pearson’s correlation coefficient. Values of *p* < 0.05 were considered statistically significant.

## 3. Results

### 3.1. Lipid Profile and VLDL and HDL Composition

VLDL and HDL were isolated from serum (Section 2.2), and the concentration of lipids and apolipoproteins in serum, VLDL and HDL was assessed (Section 2.4).

The median serum TG level for the HL group was 164 mg/dL, and it was higher compared to the NL and HC groups by 103% and 62%, respectively, on average (*p* < 0.001) (Table 1). For the HL group, the mean TC, LDL-C, and apo B concentrations were 240 mg/dL, 147 mg/dL, and 110 mg/dL, respectively, and for HC group they were 215 mg/dL, 149 mg/dL, and 105 mg/dL, respectively. These levels were significantly higher compared to the NL group (*p* < 0.001) (Table 1). There were also significant differences in serum PL, apo CII, apo CIII, and apo E levels between the groups: the highest levels were obtained for the HL group and were 259 mg/dL, 5.88 mg/dL, 16.68 mg/dL, and 5.28 mg/dL, respectively. Regarding apo CII, apo CIII, and apo E levels, the difference between the NL and the HL groups was high and amounted to 67%, 91%, and 68%, respectively (Table 1).

With regard to VLDL composition, the highest lipid (TG 139 mg/dL, TC 26 mg/dL, PL 46 mg/dL) and apolipoprotein (apo B 10.40 mg/dL, apo CII 2.87 mg/dL, apo CIII 7.29 mg/dL, apo E 1.16 mg/dL) levels were obtained for the HL group. This group was characterized by significantly higher levels of lipids and apolipoproteins compared to both the NL and HC groups (*p* < 0.001) (Table 1).

With regard to the HDL components, the HC and HL groups had significantly lower mean apo AI concentrations (166 mg/dL and 181 mg/dL, respectively) compared to the NL group (221 mg/dL). Additionally, the HL group was characterized by a statistically higher mean apo E level of 1.27 mg/dL compared to the NL group (0.86 mg/dL). In terms of the TG, cholesterol, PL, apo CII, and apo CIII levels in HDL, there were no significant differences between the groups (Table 1).

### 3.2. VLDL-TG Lipolysis Efficiency Study

VLDL was incubated with LPL in the absence and presence of HDL (Section 2.3). After incubation, the percentage of hydrolyzed VLDL-TG was evaluated, and the lipolysis efficiency was compared between the normolipidemic (NL), hypercholesterolemic (HC), and hyperlipidemic (HL) groups.

**Figure 1:**
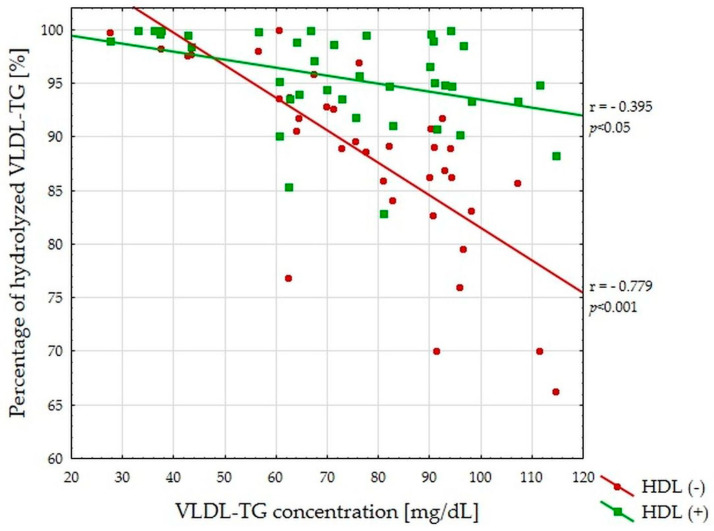
The relationship between very-low-density lipoprotein triglyceride (VLDL-TG) concentration (mg/dL) and percentage of VLDL-TG hydrolyzed during lipoprotein lipase (LPL)-mediated lipolysis for all study subjects (*n* = 40). HDL: high-density lipoprotein.

In the absence and presence of HDL, the percentage of hydrolyzed VLDL-TG in the entire analyzed group was negatively correlated with VLDL-TG concentration (Figure 1).

**Figure 2:**
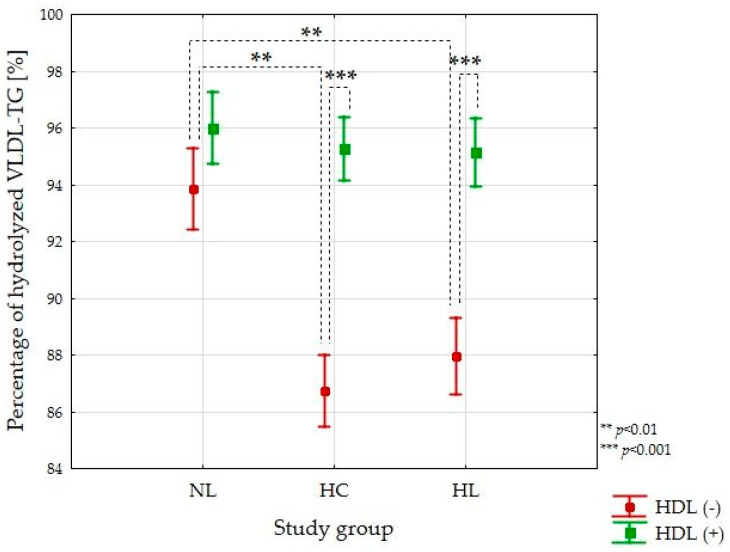
Percentage of VLDL-TG hydrolyzed during LPL-mediated lipolysis in the absence and presence of HDL obtained for normolipidemic (NL, *n* = 12), hypercholesterolemic (HC, *n* = 15), and hypelipidemic (HL, *n* = 13) groups. Data are presented as mean ± SE.

After adjustment for covariates, including VLDL-TG and age in the absence of HDL, the percentage of hydrolyzed VLDL-TG for the NL group was 94%, which was significantly higher in comparison to both the HC and HL groups (*p* < 0.01) (Figure 2). The presence of HDL significantly increased the percentage of hydrolyzed TG in the HC and the HL groups on average by ~7% (*p* < 0.001). The percentage of lipolyzed VLDL-TG in the presence of HDL was comparable between the NL, HC, and HL groups (*p* = 0.879), reaching ~95% on average.

### 3.3. VLDL Remnants Mobility Produced during Lipolysis

VLDL was incubated with LPL in the absence and presence of HDL (Section 2.3). After incubation, agarose electrophoresis of the reaction mixtures was performed, and the electrophoretic mobility of VLDL remnants was assessed and compared between the normolipidemic (NL), hypercholesterolemic (HC), and hyperlipidemic (HL) groups.

**Figure 3:**
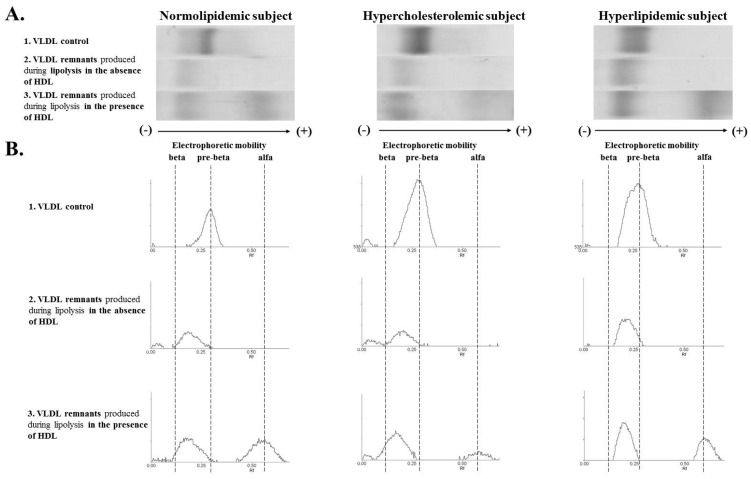
Exemplary electrophoresis (**A**) and electropherograms (**B**) of VLDL control (1) and VLDL remnants produced during LPL-mediated lipolysis in the absence (2) and presence (3) of HDL for normolipidemic (NL), hypercholesterolemic (HC), and hyperlipidemic (HL) subjects.

The relative electrophoretic mobility of control VLDLs were 0.30, 0.32, and 0.30 for the NL, HC, and HL groups, respectively (Figure 3), and they did not differ between the groups (*p* = 0.08). For all study groups, the VLDL remnants produced during lipolysis had lower relative electrophoretic mobility compared to the VLDL control between the pre‑beta characteristic for the VLDL and the beta corresponding to LDL (Figure 3).

**Figure 4:**
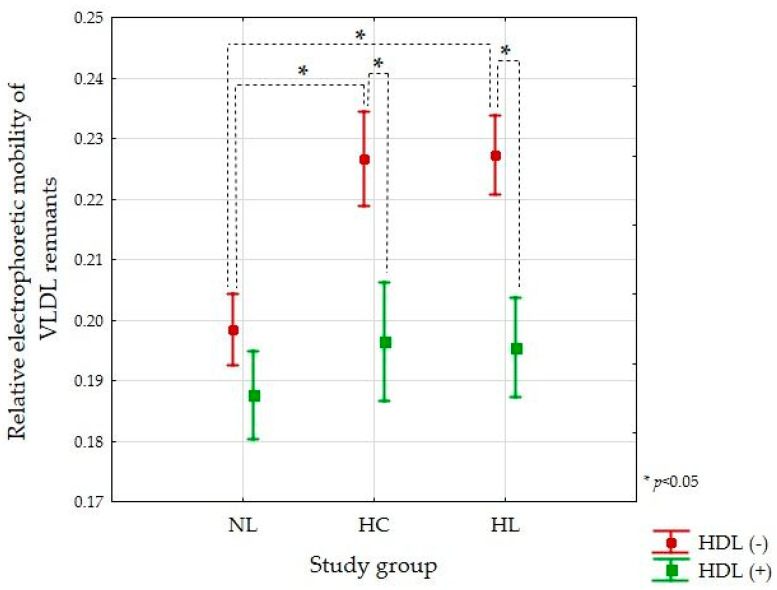
Relative electrophoretic mobility of VLDL remnants produced during LPL-mediated lipolysis in the absence and presence of HDL for the normolipidemic (NL, *n* = 9), hypercholesterolemic (HC, *n* = 6), and hyperlipidemic (HL, *n* = 7) groups. Data are presented as mean ± SE.

For the NL group, the electrophoretic mobility of the remnants produced in the absence of HDL was significantly lower compared to the HC and HL groups (*p* < 0.05) (Figure 4).

The presence of HDL influenced the electrophoretic mobility of VLDL remnants generated during lipolysis (Figure 3 and Figure 4). In the HC and HL groups, the VLDL remnants produced in the presence of HDL had significantly lower electrophoretic mobility (similar to beta) compared to the remnants produced in the absence of HDL (*p* < 0.05) (Figure 4).

There was no significant difference in electrophoretic mobility for remnants produced in the presence of HDL between the NL, HC, and HL groups (*p* = 0.732) (Figure 3 and Figure 4).

## 4. Discussion

In our study, we found that VLDL isolated from hypercholesterolemic and hyperlipidemic subjects with no history of CVD and previously untreated with hypolipidemic agents were less prone to LPL-mediated lipolysis compared to VLDL isolated from subjects with a normal lipid profile. Further, we found that the HDL was able to increase the lipolysis efficiency to the level obtained for VLDL obtained from normolipidemic subjects.

Similar to previous in vitro and postprandial lipemia studies [15,22,23], we found that one of the main factors affecting VLDL lipolysis efficiency was TG concentration. However, after adjustment for VLDL-TG level and other covariates, we observed that in the absence of HDL, there was a reduced susceptibility to lipolysis for VLDL isolated from both hyperlipidemic groups—not only from subjects with increased LDL-C and TG levels (HL group) but also from subjects with isolated hypercholesterolemia (the HC group). The lower efficiency of VLDL lipolysis was in agreement with the higher electrophoretic mobility of the VLDL remnants produced during lipolysis for these two groups compared to control, indicating less efficient conversion of VLDL into IDL/LDL.

The results of the studies concerning the impact of other factors such as TG level on VLDL catabolism efficiency are inconclusive. Van Barlingen et al. showed that VLDL isolated from normolipidemic, hypercholesterolemic, and hypertriglyceridemic subjects had similar kinetic indicators for TG lipolysis in vitro (K_M_ and V_max_) [24]. On the other hand, Chung et al. [25] observed that VLDL obtained from subjects with familial dysbetalipoproteinemia were more resistant to in vitro conversion by LPL into particles having LDL-like density compared to VLDL obtained from normolipidemic subjects.

Some data from postprandial lipemia studies suggest that VLDL catabolism disturbances can be associated with hypercholesterolemia and not increased TG levels. Tiihonen et al. [26] showed that obese subjects with an elevated LDL-C concentration and with no significant TG increase had a statistically higher maximum concentration (C_max_) of postprandial TG and had a prolonged time to achieve C_max_ over normolipidemic subjects, suggesting that the prolonged postprandial TG response in obese hypercholesterolemic subjects could be caused by impaired and delayed VLDL metabolism related to hypercholesterolemia. In FH subjects, the prolonged TG response after a fatty meal and delayed TG clearance was also reported [16]. Enhanced postprandial lipemia in hypercholesterolemic subjects compared to the control group was also observed by Cortes et al. [27]. A tendency to basal (fasting) higher TG concentration in hypercholesterolemic patients has also been observed, which coincides with the tendency observed by Cabezas et al. [28]. The results of our study show that VLDL obtained from hypercholesterolemic but asymptomatic for CVD patients are resistant to LPL-mediated lipolysis, similar to the VLDL obtained from hypercholesterolemic and hypertriglyceridemic subjects (HL group). This indicates that hypercholesterolemia can be related to disturbances in VLDL plasma catabolism and HTG development.

The resistance of VLDL to LPL-mediated lipolysis can be related to its size and/or composition [29]. In our study, we observed that VLDL particles isolated from hyperlipidemic subjects had increased lipid and protein levels compared to normolipidemic subjects. Apart from the elevated VLDL-TG concentration, subjects with elevated TG and LDL-C levels (HL group) also had significantly higher cholesterol and PL levels in VLDL compared to normolipidemic subjects. These subjects also had a higher concentration of apolipoproteins playing a key role in VLDL lipolysis; apo CII, apo CIII, and apo E. Apo CII activate LPL [30], and apo CIII is an LPL inhibitor [31]. However, in hyperlipidemic subjects, increased apo CII [32] and apo CIII [33] concentrations in VLDL were observed. Earlier studies show that apo CII level can be increased in patients with obesity or accumulation of TG-rich lipoproteins in plasma [34], and it has also been recently shown that the apo CII:apo CIII ratio was not associated with apo B-100 or plasma TG levels [34]. In our study, we did also not observe the differences in the apoCII:apoCIII ratio between the groups. Thus, an elevated apo CII level in hyperlipiedmic subjects may represent a compensatory effect in HTG to prevent further increases in TG [35]. Apo CIII and apo E play a documented role in inhibiting lipolysis and increasing TG concentration [36]. Increased levels of apo CIII and apo E in VLDL obtained from patients with hypertriglyceridemia were also described earlier [33,36]. An elevated concentration of lipolysis inhibitors plays an important role in impaired VLDL catabolism. Tomiyasu et al. analyzed the metabolism of VLDL and IDL, which were isolated according to their apo E content in normolipidemic women and demonstrated that VLDL-apo E (+) had a lower fractional catabolic rate (FCR) and higher apo CIII concentration than VLDL-apo E (−). Additionally, the rate of lipolysis of VLDL and its conversion into IDL was greater for VLDL-apo E (−) than for VLDL apo E (+) [37]. In later studies, Mendivil et al. showed using apo B lipoprotein fractions separated by apolipoprotein composition and density that VLDL-apo CIII (−) was characterized by faster clearance than VLDL-apo CIII (+). Moreover, VLDL-apo CIII (+) rich in apo E had lower clearance than VLDL apo CIII (+) poor in apo E [38].

With regard to hypercholesterolemic subjects with no increase in TG level (the HC group), we did not find a statistically significant difference in TG, cholesterol, and PL concentration in VLDL compared to the NL group. This suggests that observed VLDL metabolism disturbances may precede significant changes in lipoprotein composition.

The presence of HDL significantly increased the efficiency of VLDL lipolysis for the HC and HL groups. A similar positive HDL impact on lipolysis efficiency was perceived in our previous research [15]. We also noted earlier that phosphatidylcholine liposomes could increase the extent of VLDL surface material released during lipolysis and contribute to greater decay of VLDL-TG [39]. This confirms the assumption that PL-rich particles, such as HDL, have the ability to accept surface material released from VLDL during lipolysis and can intensify the VLDL lipolysis process. However, for the NL group, we did not observe a positive effect of the presence of HDL on LPL-mediated lipolysis efficiency. It was most likely caused by the high baseline (in the absence of HDL) lipolysis ratio—94% versus 95% of hydrolyzed TG in the absence or presence of HDL, respectively. Similarly, no effect of HDL on VLDL lipolysis in normolipidemic individuals was observed by Chung et al. [40].

The results of the quantitative TG measurements in the lipolysis efficiency study were confirmed by the electrophoresis results. The method of electrophoretic separation of the lipoproteins turned out to be a very good tool for assessing the degree of conversion of the VLDL fraction depending on the LPL activity. For hypercholesterolemic and hyperlipidemic groups, the presence of HDL caused a significant reduction in the electrophoretic mobility of the VLDL remnants. It corroborates the evidence of intensified catabolism of VLDL in the presence of HDL. Moreover, we did not notice the impact of HDL on the VLDL remnants’ electrophoretic mobility in the NL group, which is in agreement with the similar percentage of hydrolyzed TG in the absence/presence of HDL for this group. The change in electrophoretic mobility of VLDL remnants shows that in lipid disorders, the presence of HDL is necessary for the complete metabolic conversion of pre-beta lipoproteins (VLDL) to beta lipoproteins (LDL).

This study had some limitations. There was a relatively small number of participants included in the study, and normolipidemic subjects were on average 10 years younger than the hypercholesterolemic and hyperlipidemic subjects. This was due to a limited number of middle-aged normolipidemic subjects with no lipid disturbances or disorders that were exclusion criteria in our study. On the other hand, hypolipidemic treatment is instituted quickly to prevent the progression of lipid disturbances and CVD development, which limits the possibility of including middle-aged subjects with untreated hyperlipidemia in the study. In order to reduce the influence of differences in age on the obtained lipolysis study results, we used ANCOVA with age as a covariate. Moreover, in our study, participants had TG and LDL-C levels up to 390 mg/dL and 240 mg/dL, respectively; thus, we could not assess the disturbances of VLDL catabolism and the effect of HDL on VLDL lipolysis in subjects with more severe lipid disorders. Hence, further studies with a greater number of subjects and with more progressive lipid disorders are needed to evaluate whether HDL still has such a beneficial effect on VLDL lipolysis in this group of patients, resulting in efficient VLDL catabolism.

## 5. Conclusions

The results from our study showed that LPL-mediated VLDL lipolysis was disturbed in young and middle-aged subjects asymptomatic for CVD but with lipid disturbances—not only in those with elevated TG and LDL-C concentration but also in those with isolated hypercholesterolemia. HDL was able to increase the efficiency of VLDL lipolysis in subjects with an abnormal lipid profile, but it should be noted that dysfunction of HDL might impair this activity.

It can be concluded that the reduction in VLDL susceptibility to lipolysis in hypercholesterolemic subjects adversely affects TRL metabolism. It can also be an additional risk factor for HTG development and consequently increase the CVD morbidity and mortality risk. The results of our study provide additional evidence for the need to implement hypocholesterolemic treatment as soon as possible, even in asymptomatic CVD patients at the early stages of lipid disturbances.

## Figures and Tables

**Table 1:** Characteristics of study groups and lipid and apolipoprotein levels.

Parameter	Normolipidemic (NL) Group	Hypercholesterolemic (HC) Group	Hyperlipidemic (HL) Group	*p*-Value
Number	12	15	13	-
Age (years)	31 ± 7	40 ±13	41 ± 11	0.050 ^a^
BMI (kg/m^2^)	28 ± 5	28 ± 5	28 ± 4	0.935 ^a^
Gender (Female/Male)	8/4 (67%/33%)	6/9 (40%/60%)	6/7 (46%/54%)	0.366 ^c^
Hypertension	1 (8%)	5 (33%)	3 (23%)	0.302 ^c^
Smoking	3 (25%)	2 (13%)	3 (23%)	0.766 ^c^
**Serum lipids and apolipoproteins**				
Triglycerides (mg/dL)	81 (72–114)	101 (75–135)	164 (151–238) ^d,e^	<0.001 ^b^
Total cholesterol (mg/dL)	180 (169–188)	215 (197–239) ^d^	240 (210–258) ^d^	<0.001 ^b^
LDL-cholesterol (mg/dL)	101 ± 8	149 ± 35 ^d^	147 ±33 ^d^	<0.001 ^a^
Phospholipids (mg/dL)	227 (203–249)	232 (210–254)	259 (234–291) ^d,e^	0.007 ^b^
Apo B (mg/dL)	80 ± 13	105 ± 16 ^d^	110 ± 18 ^d^	<0.001 ^a^
Apo CII (mg/dL)	3.53 ± 1.21	4.24 ± 1.41	5.88 ± 1.43 ^d,e^	<0.001 ^a^
Apo CIII (mg/dL)	8.56 (5.87–12.21)	10.22 (7.73–11.22)	16.68 (13.50–18.53) ^d,e^	<0.001 ^b^
Apo E (mg/dL)	3.15 ± 1.03	3.79 ± 0.84	5.28 ± 1.41 ^d,e^	<0.001 ^a^
**VLDL lipids and apolipoproteins**				
Triglycerides (mg/dL)	53 ± 20	60 ± 28	139 ± 40 ^d,e^	<0.001 ^a^
Cholesterol (mg/dL)	10 (6–11)	13 (7–18)	26 (24–32) ^d,e^	<0.001 ^b^
Phospholipids (mg/dL)	16 ± 5	22 ± 12	46 ± 17 ^d,e^	<0.001 ^a^
Apo B (mg/dL)	4.78 (3.15–6.03)	6.00 (3.25–7.43)	10.40 (9.50–14.95) ^d,e^	<0.001 ^b^
Apo CII (mg/dL)	0.94 ± 0.46	1.28 ± 0.62	2.87 ± 1.21 ^d,e^	<0.001 ^a^
Apo CIII (mg/dL)	2.38 ± 1.01	3.01 ± 1.33	7.29 ± 3.72 ^d,e^	<0.001 ^a^
Apo E (mg/dL)	0.51 ± 0.17	0.55 ± 0.27	1.16 ± 0.73 ^d,e^	0.001 ^a^
**HDL lipids and apolipoproteins**				
Triglycerides (mg/dL)	16 (12–19)	12 (11–15)	15 (14–21)	0.157 ^b^
Cholesterol (mg/dL)	61 ± 15	52 ± 11	51 ± 15	0.122 ^a^
Phospholipids (mg/dL)	164 ± 39	133 ± 32	136 ± 37	0.119 ^a^
Apo AI (mg/dL)	221 ± 48	166 ± 29 ^d^	181 ± 40 ^d^	0.011 ^a^
Apo CII (mg/dL)	2.13 ± 0.89	2.44 ± 0.77	2.33 ± 0.68	0.652 ^a^
Apo CIII (mg/dL)	6.79 ± 3.56	6.36 ± 2.18	6.65 ± 2.34	0.928 ^a^
Apo E (mg/dL)	0.86 ± 0.27	1.05 ± 0.20	1.27 ± 0.50 ^d^	0.045 ^a^

NL group: low-density lipoprotein cholesterol (LDL-C) < 115 mg/dL, triglyceride (TG) < 150 mg/dL; HC group: LDL-C ≥ 115 mg/dL, TG < 150 mg/dL; HL group: LDL-C ≥ 115 mg/dL, TG ≥ 150 mg/dL. Data are presented as mean ± SD or median (twenty-fifth–seventy-fifth percentiles); ^a^ *p*-value obtained by ANOVA with Tukey post-hoc test; ^b^ *p*-value obtained by Kruskal–Wallis with Dunn’s post-hoc test; ^c^ *p*-value obtained by Chi-square test; ^d^ *p* < 0.05 vs. NL group; ^e^ *p* < 0.05 vs. HC group.

## Data Availability

The data presented in this study are available on request from the corresponding author.
